# Securing a future for responsible neuromodulation in children: The importance of maintaining a broad clinical gaze

**DOI:** 10.1016/j.ejpn.2016.04.019

**Published:** 2017-01

**Authors:** John Gardner

**Affiliations:** Science and Technology Studies Unit, Department of Sociology, University of York, York, YO10 5DD, United Kingdom

**Keywords:** Responsible research and innovation, Ethics, Deep brain stimulation, Patient-centred medicine, Bioethics

## Abstract

**Aim:**

This perspective paper provides an overview of several key tensions and challenges within the social context of neuromodulation, and it suggests a means of securing the future of paediatric neuromodulation in light of these.

**Results:**

Tensions and challenges relate to: the considerable clinical and economic need for new therapies to manage neurological diseases; significant commercial involvement in the field; funding pressures; public perceptions (particularly unrealistic expectations); and the emerging Responsible Research and Innovation initiative. This paper argues that managing these challenges and tensions requires that clinicians working within the field adopt what could be called *a broad clinical gaze*. This paper will define the broad clinical gaze, and it will propose several ways in which a broad clinical gaze can be – and indeed is being – operationalised in recent advances in neuromodulation in children. These include the use of multidisciplinary and interdisciplinary clinical team structures, the adoption of clinical assessment tools that capture day-to-day functionality, and the use of patient registries.

**Conclusion:**

By adopting a broad clinical gaze, clinicians and investigators can ensure that the field as a whole can responsibly and ethically deliver on its significant clinical potential.

## 1 Introduction

The emerging area of paediatric neuromodulation represents an exciting and highly promising set of developments in the management of neurological illness in children. However as with many emerging, innovative clinical developments, its potential may be hindered by challenges in the wider social and political context of healthcare research and provision. It is therefore necessary that clinicians and investigators within the area of paediatric neuromodulation are attentive to such challenges, and that they collaboratively establish a set of responsible practices for mitigating them. The work of the Irving Cooper (1922–1985) serves as a useful introductory illustration as to why this is important. Cooper was a pioneer in functional neurosurgery, developing several novel techniques for managing movement disorders and epilepsy: a cryosurgical probe to conduct thalamectomy,^1^ cerebellum stimulation to manage spasticity and cerebral palsy,^2^, ^3^, ^4^ and deep brain stimulation (DBS) of the thalamus or internal capsule to manage tremor, spasticity, and dystonia.^3^, ^5^, ^6^ These techniques, Cooper claimed, delivered meaningful improvements to patients. Yet during his time and until this day, his work has been treated with a mixture of admiration and scepticism. He clearly made important contributions to understandings of movement disorders and neuromodulation, but because of what has been described as his poor research technique and his inclination for working within his own, isolated “investigative domain”, the true clinical implications of his techniques cannot be elucidated.^7^ At that time, many of the standard movement disorder clinical assessment tools did not exist, and Cooper tended to rely on his own subjective measures for capturing clinical outcomes. Much of his work was published in the form of anecdotal reports rather than as part of a scientific series. Because of this, a valuable opportunity to produce a useful body of data on neuromodulation was missed, and the field as a whole was deprived of some much needed-direction.

Since Cooper's time the field of neuromodulation has been assisted by various technological developments (most markedly in imaging technology) accompanied by major advancements in understandings of neuro-networks, particularly in basal ganglia function (e.g.^8^). Clinicians now also have access to standardised, validated tools for rating disease severity and assessing clinical outcomes that enable a common language between clinical centres and the pooling of data. The field, then, is on much firmer ground that it was in Cooper's time. It is important, however, not to become complacent about these scientific and technical developments and assume they will assure that neuromodulation will live up to its considerable potential. Now more than ever it is vital that clinicians and investigators collaboratively establish a set of responsible practices for the field. There are, as this paper will explain, various contextual social, economic, and institutional factors that could hinder the field, and which pose significant challenges for biomedical innovation more generally. These relate to the commercial climate and public perceptions, pressure on national healthcare budgets, and the European Commission's call for Responsible Research and Innovation (RRI). These factors should not be considered secondary to the scientific and clinical challenges of translational medicine – they are challenges that need to be addressed and managed at all stages of the innovation process.

This paper will provide an overview of these challenges, and in doing so, it will suggest that managing these challenges will require that investigators and clinicians perceive disease as biopsychosocial phenomenon, and it will require capturing and evaluating the social impact of disease, and of the intervention. It will require, in other words, that investigators and clinicians deploy what could be called *a broad clinical gaze*. Drawing on the French philosopher Michel Foucault's notion of the Medical gaze, this paper will define the broad clinical gaze, and it will propose several ways in which a broad clinical gaze can be – and indeed is being – operationalised in recent advances in neuromodulation (some of which feature in this special issue). These include the use of multidisciplinary and interdisciplinary clinical team structures, the adoption of clinical assessment tools that capture day-to-day functionality, and the use of patient registries. Such measures, it is argued, can help with the production of a pool of valuable evidence on the clinical effectiveness and social impact of neuromodulation, and they can help ensure that innovation within the field is directed towards genuine social and clinical need. This paper will also highlight some of the institutional constraints that can hinder the ability to operationalise a broad clinical gaze. Overcoming such constraints will require coordinated action within field, and this paper will conclude by suggesting that such coordinated action can be seen as moulding an institutional context that embodies responsible research and innovation and will enable neuromodulation to deliver on its considerable clinical potential.

## 2 The social context of neuromodulation: key challenges and tensions

First and foremost, the social context of the field is characterised by a considerable and urgent need for therapies for managing neurological diseases in children. Neurological disease is a cause of great suffering for patients, and is often a huge burden for families and carers. For this reason, the Nuffield Council on Bioethics (an influential ‘think tank’ in the UK) has stated that society has a moral obligation – in accordance with the ethical principle of beneficence – to explore and develop new therapeutic interventions, and they explicitly identify neuromodulation as a promising field in this regard.^9^ Additionally, neurological illness also constitutes a huge economic burden for nation-states. According to one estimate, the total economic cost of brain disorders (which includes both mental and neurological disorders) in Europe for 2010 was 798 billion EUR; a figure which takes into account both direct costs and indirect costs (such as lost participation in workforce).^10^ This is an average cost per inhabitant of around five and a half thousand euros. More specifically, neuromuscular disorders (excluding multiple sclerosis and Parkinson's) have an economic cost of around 7.7 billion EUR and epilepsy (both adult and paediatric) has a cost of around 13.8 billion EUR.^10^ As we see further on in this section, such economic considerations are increasingly shaping health and research policies in the EU. Together, the economic implications and unmet clinical need necessitate urgent research into, and development of, neuromodulation therapies, and thus provide an important moral justification for advancing the field of paediatric neuromodulation as a whole.

This great need has of course attracted considerable commercial interest, and some therapeutic areas in the field now represent lucrative markets for device manufacturers; this is another important aspect of the social context of neuromodulation. In 2014, the total net sales of neuromodulation technology of the top three manufacturers (Medtronic, Boston Scientific, and St Jude Medical) was just under three billion USD, and generally the field is characterised by a high rate of innovation.^11^ One reason manufacturers have been so interested in the field is that the same technology platform can be adapted into multiple disease areas – this is particularly the case with deep brain stimulation,^12^ which has seen the recent dissemination of DBS technique into various psychiatric disorders. Neuromodulation typically entails complex, high-performance technology, and the regulatory burden in some therapeutic applications is high; the field, then, is dependent on industrial partners with sufficient technical and regulatory expertise and with the financial resources required to gain market access. Indeed, historically, neuromodulation (as with developments in medicine more generally) owes its existence to key clinician-industry partnerships, and manufacturers have been instrumental in facilitating the dissemination of the technology into new disease areas.^13^ The dependence on industry need not be seen negatively, but some commentators have argued that the considerable commercial interests within the area necessitate that investigators and clinicians maintain a degree of vigilance.^14^ Corporate motivations could, it has been suggested, unnecessarily put patients at risk, and specific concerns have been raised in the US regarding a manufacturer's supposed ‘misuse’ of a regulatory exemption in DBS for adult obsessive compulsive disorder.^15^ Such sentiment no doubt reflects a more general distrust of large industry involvement in healthcare that has been stoked by past scandals in the domain of pharmaceuticals, but regardless as to whether such sentiment is justified, it is expedient for investigators within the field of neuromodulation to be aware of, and reflect upon, the influence of commercial interests. More specifically, it is wise for investigators in paediatric neuromodulation to adopt an approach to clinical research that ensures that commercial interests align with those of the patient, and that the latter are not ignored in the pursuit of lucrative markets.

Funding pressures represent another key aspect of the social context of neuromodulation. Rising healthcare costs, driven partly by the introduction and dissemination of high-cost technologies, is a major concern for governments. Healthcare budgets are under increasing strain, and there is considerable political pressure to cut costs and find efficiencies within healthcare systems. In the UK, for example, the NHS is said to be heading towards the equivalent of a 40 billion EUR funding gap by 2021.^16^ One consequence of this is that commissioning authorities are increasingly relying on formal technology appraisals when making decisions as to whether a new medical therapy will be implemented within a healthcare system. Within the EU, initiatives have been launched to encouraging a convergence towards the adoption of health technology assessment (HTA) standards for medical devices: the EU Commission-funded MedtechHTA project, for example, aims to identify appropriate HTA methodologies, and to encourage greater harmonisation among the HTA authorities of member states.^17^

This means that it is becoming increasingly important to demonstrate the cost effectiveness and clinical effectiveness of new device-based therapies. The general purpose of an HTA is to determine: to what degree does the therapy benefit the patient? What is the cost of delivering this benefit to all those in the proposed patient group? What are the wider social benefits of the therapy? And in some cases: how does the benefit/cost compare to existing standards of care? Currently the ways in which these questions are addressed can vary greatly from one HTA authority to another, but in most cases the data required to do so is much broader in nature than that which is required to obtain regulatory approval. The determination of patient benefit (or clinical effectiveness), for example, may require a broad array of data (both quantitative and qualitative) on the impact of the therapy in the patient's day-to-day life, and the calculation of ‘cost effectiveness’ can entail a variety of data including the direct costs (setting-up supporting infrastructure, training staff, etc), and indirect costs (such as those resulting from changes in work productivity and social care). Manufacturers and investigators are thus expected to design clinical studies in such a way that this various data can be collected.

The relevance of HTA to the field of paediatric neurostimulation will obviously differ depending on the specific application. Low cost devices, or devices used in clinical studies or in ‘one-off’ treatments, will continue to be commissioned at a regional level by hospital authorities. However, therapies that are perceived to have high-up front costs or a significant target population, are more likely to be subject to formal HTA. Manufacturers will be expected to clearly demonstrate the cost implications and the clinical benefits compared to existing standards of care. In other fields such as regenerative medicine, HTA has been labelled a major hurdle to clinical adoption, and manufacturers are being encouraged to cater their product development pathways accordingly as early as possible.^18^

Public perception, particularly hype and high expectation, is another important aspect of the neuromodulation social context that needs to be considered. It is not unusual for news media to propagate highly optimistic and potentially misleading representations of biomedical advancements, using terms such as ‘breakthrough’, ‘life-changing’, and ‘revolutionary’.^19^ Much social science work has explored the consequences of this.^20^, ^21^ On the one hand, such optimism provides biomedical projects with much needed momentum – it helps attract and consolidate the attention, resources and investment necessary for further development. On the other hand, optimistic portrayals can exacerbate distrust and scepticism among the public, especially when projects fail to deliver on their promise.

Hype and high expectation has surrounded developments in neuromodulation, particularly deep brain stimulation. Several studies have explored media coverage of DBS for adult movement disorders, noting that it has been ‘overly optimistic’ and has tended to focus on those individual cases that do dramatically well, thus giving the impression that all patients will respond in this way.^22^, ^23^ Clinicians working within DBS paediatric services have reported that families often arrive with unrealistic expectations, and that managing these expectations entails considerable work.^24^ Such work is a necessary aspect of the informed consent process, but it is also necessary for protecting the reputation of the service and the therapy itself. Clinicians have felt that if family expectations are not sufficiently managed and families (who are often active in social media) feel mislead, they are “setting their service up for certain failure”.^24^ It is important, then, that clinicians careful scrutinize the expectations of patients and their families, and communicate the likely benefits of neuromodulation therapies using accessible terms and frames of reference. This is of course particularly important in highly invasive procedures such as DBS.

It is partly in response to these tensions surrounding biomedical innovation (and science and innovation more broadly) that the European Commission launched its Responsible Research and Innovation (RRI) initiative.^25^ The motivation for RRI has been a perception among European policy makers that existing policy is inadequate for guiding innovation in emerging, potentially ethically problematic areas of science and technology.^26^ The initiative is still very much in its infancy, and the details of what exactly qualifies as ‘responsible’ have not been clearly delineated, but the overall purpose of the initiative is to encourage research and innovation that addresses the needs of European citizens in a manner that accords with European values. Advocates argue that research and innovation projects need to be responsive to the input of a range of stakeholders who can represent and identify the needs of citizens^25^: greater public engagement that broadens input beyond commercial and professional interests can help ensure that innovation is better directed towards genuine social need.

The RRI initiative is intended to guide the formation of national and European-level policy. Exactly what impact it will have it is not clear, but it is likely that funders and other influential bodies will expect investigators to demonstrate sensitivity to the core elements of RRI. Indeed, the Nuffield Council on Bioethics has adopted the initiative into their ethical framework for developing novel neurotechnologies^9^: They argue that RRI in the context of neurotechnology (within which they include neuromodulation) would entail the generation of robust evidence on safety and efficacy, constant reflexive evaluation of the specific technology, and the input of multiple stakeholder perspectives.^27^ RRI represents a significant and potentially influential discourse in the social context of paediatric neuromodulation, and indeed, biomedicine more generally. It is important, then, that activities of investigators and clinicians within the field reflect an awareness of the initiative.

The following section will introduce a means by which clinicians working within paediatric neuromodulation can both adhere to RRI and navigate the other challenges outlined above. This is to adopt what will be described as a *broad clinical gaze*. In general terms the broad clinical gaze is a particular attitude to clinical work, but as Section 3 will suggest, there are specific means by which the broad clinical gaze can be operationalised in clinical practice.

## 3 The broad clinical gaze: enacting responsible research and innovation

The tensions and challenges outlined above have set forth a set of requirements for clinicians and investigators working within emerging areas of biomedicine such as paediatric neuromodulation. First, it is becoming increasingly important to ensure that the viewpoints of citizens are incorporated into the design of clinical studies and the design of clinical services providing novel treatments. Generally, this means the viewpoints of patient association and advocacy groups. Second, clinical studies and clinical services providing novel therapies, particularly those that are high cost, need to generate data that can be used to calculate cost effectiveness. And third, clinical studies and services also need to be able to generate data on the benefits of a therapy. This includes data on the day-to-day social impact of the therapy for the child and their family, which is wider in scope than that which is generally used to demonstrate clinical ‘efficacy’. If clinicians and investigators are able to sufficiently satisfy each of these requirements, then it will be possible to objectively elucidate the true benefit of neuromodulation using criteria that align with the values and perspectives of those who will be most affected by it. To do this, I propose that it is expedient for clinicians and investigators to adopt a broad clinical gaze. In order to explain precisely what this is and how it can be operationalised in clinical practice, it is necessary to briefly introduce the concept of the *medical gaze*, as it has been characterised by the highly influential French philosopher Michel Foucault.^28^

The medical gaze, Foucault argued, is a particular way of perceiving disease and the body that emerged in the early 19th century, and which has since become the basis of modern medicine. Prior to its emergence, disease was perceived to be a phenomenon that affected the physical body, but ultimately existed in an immaterial ‘spiritual’ realm. The emergence of the medical gaze signalled a paradigm shift; a reconceptualization of disease as a material, physical phenomena – bio-physiological processes – that could be understood through close empirical investigation of the diseased-body.^28^ With this change in perception medicine became a ‘true science’ and flourished, and the patient's body became an invaluable source of biomedical data. The emergence of the medical gaze is thus associated with what has been referred to as the biomedical model of disease.^29^ The positive impact of this paradigm shift is immense and incalculable. Nevertheless it did recast the clinician–patient relationship in such a way that it acquired, in some contexts, an ethically-problematic paternalistic dynamic: The clinician's viewpoint, informed by their expertise in reading biomedical signs and symptoms, eclipsed that of the patient who experienced the disease, and in some contexts this meant that patients had very little input in decisions regarding their care.^30^ Since the 1970s advocates of patient-centred medicine – which has influenced healthcare policy in many countries^31^ – have lobbied for greater accommodation of patient viewpoints in healthcare practices, and they have argued that disease should be perceived and treated as a biopsychosocial phenomenon, rather than just a biomedical one.^32^ It is within this line of thinking that the notion of broad clinical gaze has its genesis: it is a broadening of the medical gaze described by Foucault^28^ to include psychological and social aspects of disease. It can therefore be defined as:

*A clinical interest that extends from the shapes and structures of the body, to the emotional state of the patient, to elements of the patient's social context and their ability to act within it.*

Modern medicine, brought about by the emergence of the medical gaze, has had an immeasurable impact on human welfare. However, medical innovation in the current social context increasingly necessitates that clinicians and investigators adopt a broader perspective of disease: a broad clinical gaze, thus recognising that diseases have important non-biomedical aspects which need to be accommodated in the design of clinical studies and clinical services providing novel therapies.

Given that the patient-centred medicine movement has already influenced a great deal of clinical work in many contexts, the broad clinical gaze will not represent a stark departure from how many clinicians and investigators approach their day-to-day work. This is especially true for those working in the field of paediatric neuromodulation: clinicians working in paediatric settings have generally been concerned with, and attentive to, the social impact of childhood chronic illness, particularly the impact on family life. As a general philosophy, then, the broad clinical gaze will align with current, common perspectives on how clinical research should be conducted and how clinical services should be delivered: indeed, in the following section I highlight some existing practices and examples – some of which are reported in greater depth in this special issue – of how the broad clinical gaze can be operationalised in clinical work.

### 3.1 Operationalising the broad clinical gaze

One effective way of operationalising the broad clinical gaze in clinical settings is via multidisciplinary and interdisciplinary teamwork. Multi- and interdisciplinary teams contain a range of professional viewpoints which can help provide a more holistic understanding of the impact of disease upon a patient and the impact of an intervention. The ideal composition of a team will vary depending on the nature of the disease and the intervention being offered. Small teams are suitable for delivering routine, low risk (non- or minimally-invasive) neuromodulation therapies, but invasive, high-cost therapies such as DBS for movement disorders may be better delivered by larger teams representing a range of professions including allied health disciplines (physiotherapy, clinical psychology, speech and language therapy), and ideally disciplines that place a heavy emphasis on patient-centred approaches (such as occupational therapy). The collective expertise of such teams enable them to scrutinize and attend to the biomedical, psychological and social (or domestic) aspects of neurological disease, and to examine how these various dimensions are affected by neuromodulation. While each member of a team will be working towards the same broad goals (improving the well-being of patients and their families), tensions may arise as team members draw on their specific disciplinary-based understandings and values. Such tension need not be seen negatively; indeed, it can prompt teams to engage in reflexive evaluation – an important component of RRI, according to the Nuffield Council on Bioethics.^9^ It is important, however, that team members are provided with regular opportunities to provide meaningful input into discussions and clinical decision-making^33^: studies of teams in other fields have noted that the potential benefits of interdisciplinarity can been stunted by traditional hierarchies as doctors – with their largely biomedical-based clinical opinion – dominate clinical decision-making^34^

Another advantage of such teams is that allied health professionals may have more opportunities to spend time with families, perhaps as they conduct assessments. This can facilitate trust and open communication, and families will be more willing to speak openly and willingly about their challenges and their expectations. Indeed, multi & interdisciplinary teams are well-suited to managing the expectations of patients and families. Drawing on discipline-specific expertise, they can elucidate the hopes and expectations of families and communicate anticipated clinical outcomes to families, using frames of reference that patients and families can understand, such as an improved ability to perform specific activities of daily living.^24^

Another way in which the broad clinical gaze can be operationalised in neuromodulation is via the adoption of patient-centred clinical assessment tools. Such tools, which are commonplace in allied health disciplines (particularly occupational therapy) can be used to capture clinical changes that families themselves feel are important. Assessment tool that capture and quantify the psychological and social impact of disease can be used to complement the more conventional impairment based tools commonly used in neurology. Some examples include the Canadian Occupational Performance Measure^35^ which measures patients' self-perceptions of their abilities, and the Assessment of Motor and Process Skills^36^ which can be used to assess a patient's ability to perform domestic tasks (‘activities of daily living’) that patients and families would like to improve. Both tools can provide clinicians with a sense of how the disease and the intervention are impacting day-to-day life. The importance of using such tools as part of a broad assessment regime has been demonstrated by clinicians working within a service providing DBS to young patients with dystonia (Gimeno, this issue). These patient-centred tools appear to capture important improvements that impairment based measures (the Burke-Fahn-Marsden Dystonia Rating Scale) are insensitive to, particularly among patients with complex secondary dystonias.^37^ The data that patient-centred tools produce support reflective evaluation of the neuromodulation intervention, and they may be essential for calculating cost and clinical effectiveness of the intervention as part of a formal HTA.

Patient registries, such as the Dystonia DBS database currently under construction and reported here in this issue, are another means by which the broad clinical gaze can be operationalized. Patient registries and databases can have a variety of purposes (documenting the natural history of a disease; providing an inventory of patients for clinical research etc) but they can be a particularly useful tool for capturing data on the impact of an intervention, especially in contexts where larger scale clinical trials are not possible and when the intervention is being offered in a few dispersed centres.^38^, ^39^ Depending on how a registry is designed, it can capture data relating to the cost and social impact of an intervention. It may, then, provide valuable evidence for formal health technology appraisals^40^: the UK's National Institute for Health and Care Excellence (NICE), for example, has used data captured in registries for conducting cost effectiveness evaluations in other fields.

A particularly important feature of registries and databases is that they provide an opportunity for patient-involvement in research design. Patient associations and affected families can be consulted in the design of registries to ensure their concerns are reflected in the type of data that is collected. Patient association groups may also be involved in the day-to-day management of the registry.^37^ They can, then, serve as a useful means of ensuring that the interests and values of citizens are being attended to in neuromodulation research and delivery, thus facilitating responsible research and innovation within the field. The need for a database to capture the impact of DBS in paediatric dystonias has been recognised by clinicians in the field – in this issue, Marks reports on efforts to produce a database that will pool together DBS outcomes from several centres, and will thus serve as a useful body of evidence on the effectiveness of DBS.

There are, of course, considerable challenges in establishing patient registries and databases. They require constant oversight and long-term funding. Care needs to be taken during their design to ensure that the data they collect can address key, well-defined objectives. Clinical centres will need to decide on a set of shared standards for collecting data for the registry, and ideally, registries will be designed so that additional, perhaps unanticipated concerns can be addressed in the future. There is, then, a great deal of work and negotiation involved in the establishment and maintenance of patient databases. Partly in recognition of these challenges, the European Commission is co-funding actions to promote and support the establishment of patient registries within EU member countries.^41^

## 4 Conclusion: Institutionalising RRI within paediatric neuromodulation

Here I have identified several ways in which the broad clinical gaze can be – and indeed has been – operationalized in clinical practice within the field of neuromodulation: Multidisciplinary teamwork, the adoption of patient-centred clinical assessment tools, and the use of patient registries are useful means by which clinicians working within neuromodulation can navigate the tensions described above and align their field with the tenets of responsible research and innovation. This is, of course, easier said than done. Inevitably, institutional constraints will hinder the ability to operationalise a broad clinical gaze in clinical settings. In many contexts, institutional funding pressures or rigid payment structures will mean that it is not financially feasible to support a multidisciplinary service containing the ideal range of health disciplines. There may be a reluctance to adopt clinical assessment tools from the allied health disciplines because journal editors and reviewers within neurology may be unfamiliar with them, preferring the use of ‘heritage’ impairment tools. Clinical teams may be reluctant to place their arduously-collected data on a shared registry or database, especially in a competitive professional climate.

The specific means of operationalised the gaze outline here – multidisciplinary teamwork, the adoption of patient-centred assessment tools, and the use of patient registries – will obviously not be appropriate for all areas of neuromodulation. It is important therefore that clinicians within the field remain attentive to other potential ways of practicing a broad clinical gaze, and other ways of adhering to the sentiments of responsible research and innovation. Are there, for example, other ways in which the social impact of a neuromodulation therapy for children can be captured? Are there other ways of engaging children, families and patient associations within research into neuromodulation, and in the design of neuromodulation services? Operationalising a broad clinical gaze within neuromodulation, therefore, will require some degree of institutional change, and this in-turn will require co-ordinated effort from those working within the field. It is for this reason that a regular forum for those working within the field, such as the recent EPNS satellite symposium (May, 2015), is necessary: it provides an opportunity for reflexive evaluation of the field as a whole, knowledge sharing, and the coordination of effort across international contexts. These activities are needed to ensure that the field as a whole can responsibly and ethically deliver on its significant clinical potential to address the huge clinical need, and they will, hopefully, mean that in forty years' time commentators will not look back on this era of neuromodulation with the same sense of frustration as we do when reflecting on the work of Irving Cooper.

## Conflict of interest statement

The author, John Gardner, certifies that he has NO affiliations with or involvement in any organization or entity with any financial interest (such as honoraria; educational grants; participation in speakers' bureaus; membership, employment, consultancies, stock ownership, or other equity interest; and expert testimony or patent-licensing arrangements), or non-financial interest (such as personal or professional relationships, affiliations, knowledge or beliefs) in the subject matter or materials discussed in this manuscript.

The paper draws on research that was funded by the Wellcome Trust (Wellcome Trust Biomedical Ethics Strategic Award 086034).
